# Determinants of Antenatal Healthcare Utilisation by Pregnant Women in Third Trimester in Peri-Urban Ghana

**DOI:** 10.1155/2018/1673517

**Published:** 2018-02-15

**Authors:** Jones Asafo Akowuah, Peter Agyei-Baffour, Dadson Awunyo-Vitor

**Affiliations:** ^1^Department of Agricultural Economics, Agribusiness and Extension, Kwame Nkrumah University of Science and Technology, Kumasi, Ghana; ^2^Department of Community Health, Kwame Nkrumah University of Science and Technology, Kumasi, Ghana

## Abstract

Access to quality healthcare still remains a major challenge in the efforts at reversing maternal morbidity and mortality. Despite the availability of established maternal health interventions, the health of the expectant mother and the unborn child remains poor due to low utilisation of interventions. The study examined the socioeconomic determinants of antenatal care utilisation in peri-urban Ghana using pregnant women who are in their third trimester. Two-stage sampling technique was used to sample 200 pregnant women who were in their third trimester from the District Health Information Management System software. Well-structured questionnaire was the instrument used to collect data from respondents. Descriptive statistics and inferential statistics including binary logit regression model were used to analyse the data with the help of SPSS and STATA software. The results showed varying utilisation levels of ANC. From the regression result, age, household size, and occupational status were identified as the important socioeconomic determinants of antenatal care utilisation among the respondents. The important system factors which influence antenatal care utilisation by the respondents are distance to ANC, quality of service, and service satisfaction. The study concludes that socioeconomic and health system factors are important determinants of antenatal care utilisation. Stepping up of interventions aimed at improving the socioeconomic status and addressing health system and proximity challenges could be helpful in improving antenatal care utilisation by pregnant women in Ghana.

## 1. Introduction

The health status of women is a major component in the socioeconomic development of societies [1–3]. The value of women's health is, therefore, paramount in societies due to the productive roles they undertake [4, 5]. According to Kickbusch [6], the quality of life of women depends on their health and education. Hence, “the healthcare that a mother receives during pregnancy, at the time of delivery, and soon after delivery is important for the survival and well-being of both the mother and her child” [7]. About 580,000 women of reproductive age die each year from complications arising from pregnancy worldwide, and almost half of these deaths occur in the Sub-Saharan Africa (WHO, 2003) [8]. Women in the Sub-Saharan Africa face a 1 : 16 life risk of dying in pregnancy and childbirth, compared with 1 : 2800 chances for women in the developed world [9, 10]. However, the Reproductive and Child Health Unit of the Ghana Health Service [11] estimates maternal mortality around 230 per 100,000 live births. In order to enforce the sustainable development goals 3 and 5, there is the need to promote women's health by helping them to increase control over and improve their health status [1, 12–14]. As a result, good health of women ensures their active involvement in all sectors of economy and increased well-being through wealth creation. A major means of improving maternal healthcare is strengthening antenatal care, which is a major component of maternal health services. This brings the expectant mother closer to the caregiver and increases her chances of survival through the use of other services that accompany the antenatal care.

Antenatal healthcare is defined by the World Health organisation (2000) as the “care a pregnant mother receives before birth” and involves among other services education, screening, counselling, treatment of minor ailment, and immunisation. Antenatal care coverage, however, is defined by Arthur [15] as the “percentage of women who use ANC services provided by skilled health personnel for reasons related to pregnancy at least once during pregnancy, as a percentage of live births in a given time period usually one year.” According to the Ghana Ministry of Health [16], the basic objective of antenatal care in Ghana is to “promote and maintain” the health status of the pregnant mother. Its main purpose is early detection and management of pregnancy-related complication [17]. Empirical studies have shown that antenatal care, a section of maternal healthcare, cushions women with the support to detect early problems associated with pregnancy and to reduce imminent labour complications [18, 19] (Doku et al., 2012). It is of this view that the Ghana Ministry of Health introduced the free maternal healthcare delivery nationwide in April 2005 of which antenatal care was a major component. The policy is expected to reduce the cost on maternal services which serves as a burden to pregnant mothers and reduces maternal mortality rate [15]. The policy in Ghana is expected to increase maternal healthcare utilisation and cost-effectiveness, and despite being universal in application, it can benefit the poor in rural areas. In spite of this intervention, several factors such as the need for adequate funding and strong institutional ownership beset the free maternal healthcare policy (Sophie et al., 2009). Others are poor infrastructure in the rural communities, lack of government commitment to the development of rural health systems, geographic isolation to rural healthcare, and poor socioeconomic characteristics of women especially the rural counterparts.

Again, social exclusion and discrimination against rural women, poor livelihood objectives and outcomes, and life expectancy being shorter with the prevalence of diseases further worsen the social gradient in rural societies. Hence, such conditions inhibit the vulnerable groups with women being the majority to access healthcare. Yet, maternal mortality is on the increase even after the policy was introduced [20]. There is the growing concern to reduce barriers to health accessibility internationally, nationally, and locally with special emphasis on vulnerable groups (Witter et al., 2009). There have been some studies on the determinants of ANC use in Ghana including those of Overbosch et al. (2005) and Abor et al. [18] who after using data prior to the introduction of the policy found significant influence of socioeconomic factors affecting the rate of ANC use. Arthur [15] who also uses data after the policy was introduced finds significant influence of socioeconomic factors with particularly wealth affecting ANC use. It is generally acknowledged that the health status of people is influenced by a variety of factors which are outside the domain of the public health system [21]. It is therefore imperative to address the origin of ill-health when maternal health is discussed [22–25]. Health differences exist because of a combination of behavioural, structural, material, and cultural factors [26]. The social context of mothers must be taken into consideration when their health is defined [3]. These studies consider the influences of ANC use at the national level and fail to consider the immediate social setting of expectant mothers. The aim of this study is to investigate if socioeconomic factors apart from wealth have effects on antenatal healthcare services in peri-urban Ghana.

## 2. Related Literature

Socioeconomic factors comprise demographic, social, structural, and attitudinal influences which increase the likelihood of a person to seek for ANC services when pregnant (Andersen 1995). Among the examples of socioeconomic variables that influence the use of ANC are age, level of education, employment status, household size, geographic distance, and physical accessibility. These factors can influence the ANC use of pregnant women either positively or negatively. The age of the pregnant mother affects her quest in the use of ANC. Available studies on age physical accessibility and ANC use reveal varied evidence. For example, a study conducted by Arthur [15] on utilisation of maternal healthcare services in Ghana reveals that antenatal care utilisation reduced with respect to age increase of expectant mothers. Also, Owili et al. [27] found a reduction in the proportion of women obtaining ANC services with increasing age in Kenya. On the contrary, studies from Navaneetham and Dharmalingam (2002); Klemetti et al. [28]; Cheng et al. [29], and Uppadhaya et al. [30] confirm that older women were more likely to use antenatal care services than their younger counterparts. The reason may be due to the fact that older women might have gathered immense knowledge on the need of antenatal care services, which may positively influence their use of ANC services.

The level of education of an expectant mother determines the utilisation level of antenatal healthcare. It is established in the available literature that the education level of pregnant women determines their antenatal care use. According to Greenaway et al. (2012), the association between maternal education and use of health services in Ghana demonstrates a strong link between mothers' formal education and a composite measure of women's health knowledge in accessing healthcare services. Other studies from APP [10]; Arthur [15]; Finlayson and Downe [31]; Anastasi et al. [32]; Agha and Williams [33]; and Sahito et al. [34] have found a positive and significant association between education and maternal healthcare use. Nigussie et al. (2004), in their work on the assessment of safe delivery service utilisation among women of child bearing age in north Gondar zone, North-West Ethiopia, discovered that educational status of mothers had significant influence on utilising safe delivery services. According to the study, 72% of women with secondary or higher education received ANC from health professionals, compared to 45% of women with primary education and 21% with no formal education. The Ghana Multiple Indicator Cluster Survey (GSS, 2011) reveals that 78 percent of women with no education received ANC for four or more times, compared to 97 percent of women with secondary or higher education. Also, only 44 percent of mothers with no education delivered with the assistance of skilled personnel, compared to 95 percent for women with secondary or higher education.

It is an established fact that the employment status of expectant women influences their ANC use. The involvement of women in employable ventures positively influences their use of quality medical care and services (Chakraborty et al., 2003) [18]. This empowers pregnant mothers to increase control over the things which affect their lives as far their healthcare needs are concerned. In their study on the socioeconomic determinants of maternal healthcare utilisation in seven countries, Saad-Haddad et al. [35] reveal that household wealth significantly influenced the facility type for accessing maternal care. Abekah-Nkrumah and Abor [36] and Rai et al. [37] also found out that household income is linked with frequent use of modern healthcare.

Household size is another important predisposing factor believed to influence the utilisation of antenatal care. Household size is measured as the number of persons in a particular household that are dependent on the pregnant mother for their daily sustenance [7, 38]. It is widely acknowledged that women with large family sizes tend to underutilise maternal healthcare services due to excessive demand of their money, time, and other resources [18]. The situation becomes worse in developing countries like Ghana when women, aside child bearing roles, spend a lot of time to fetch water and firewood, prepare daily meals, and keep the house in order. In his study on analysing the primacy of distance in the utilisation of health services in the Ahafo-Ano south district of Ghana, Buor (2003) suggests that a large household size would mean a lower average income generally in poor communities. A number of studies support the view that ANC use is significantly influenced by the household size of mothers [39, 40].

## 3. Methodology

### 3.1. Sampling and Data Collection

Two-stage sampling technique was adopted to sample the respondents for the study. At the first stage, purposive sampling technique was used to select four health facilities in peri-urban areas in Kumasi. The selection was based on the level of antenatal care attendance at the health facility. Using the District Health Information Management System software in the second stage, list of pregnant women in their third trimester was obtained from the antenatal units of the sampled health facilities and simple random sampling technique was used to select 200 pregnant women for the study. Structured questionnaires were used to collect data from the respondents. Data collected from respondents included demographic characteristics, health status, family planning services, social support systems, quality of service, choice of facility, level of utilisation, and views on caregivers. Data were coded and analysed using the SPSS and STATA (12), respectively. Since the study deals directly with respondents, ethical approval was sought from the Committee on Human Research, Publication and Ethics-KNUST, Kumasi, under the auspices of the Komfo Anokye Teaching Hospital, Kumasi.

### 3.2. Analytical Framework

The pregnant women in the third trimester may either be seen as having utilised antenatal care or not depending on number of visits resulting in a binary dependent variable *y*_*i*_. The binary dependent variable *y*_*i*_ takes on the values of zero (0) (if the number of visits to antenatal care is less than four) and one (1) (if the number of visits to antenatal care is greater than three as outlined by the WHO) [41].

The probability of observing a value of one is

(1) $$\begin{array}{c} P_{r}=\left(\;y_{i}=\frac{1}{x_{i}\beta_{i}}\;\right)=1-F\left(\;x_{i}\beta_{i}\;\right), \end{array}$$

where *F*(·) is a cumulative distribution function; it is a continuous, strictly increasing function that takes a real value and returns a value which ranges from 0 to 1. Consequently, the probability of observing the zeros is

(2) $$\begin{array}{c} P_{r}\left(\;y_{i}=\frac{0}{x_{i}\beta_{i}}\;\right)=F\left(\;-x_{i}\beta_{i}\;\right). \end{array}$$

Given the above specification, the maximum likelihood estimation approach can be used to estimate the model.

The dependent variable *y*_*i*_ is an unobserved latent variable that is linearly related to by the equation:

(3) $$\begin{array}{c} y_{i}=\beta_{i}x_{i}+\mu_{i}, \end{array}$$

where *μ*_*i*_ is a random disturbance term and *x*_*i*_ is independent variable which influence the number of antenatal visits. The observed dependent variable is determined by whether *y*_*i*_ exceeds three or otherwise:

(4) $$\begin{array}{c} y_{i}=\left\{\begin{array}{cc} 1 & \text{if }y_{i}^{*}>0\\ 0 & \text{if }y_{i}^{*}\leq 0, \end{array}\right. \end{array}$$

where *y*_*i*_^*∗*^ is the threshold value for *y*_*i*_. This study adopted the logit model to analyse the data, and the empirical model is specified as

(5) yi=∝0+∝1X1+∝2X2+∝3X3+∝4X4+∝5X5+∝6X6+∝7X7+∝8X8+∝9X9+∝10X10+∝11X11+∝12X12+ε,

where variables, their description, and their expected sign are shown in Table 1.

## 4. Results

### 4.1. Descriptive Statistics

The *z* scores adopted by the study are used to determine whether there is a significant difference in the use of ANC by the independent variables and the rate of such differences if any and the descriptive statistics of the variables used in the regression analysis are displayed in Table 2. From Table 3, the log likelihood value of −18.83 is the smallest possible deviance between the observed and predicted values. The likelihood ratio chi-square of 156.69 is significant at 1%. This indicates that the null hypothesis that a model with no independent variables (constant only model) is better than the model used for the present study was rejected. Again, Table 3 shows that out of the 19 independent variables, 10 were significant at 90% confidence interval. Age, household size, occupational status dummy, and secondary/SHS educational level are all significant at 5%. The satisfaction dummy, quality of service dummy, Asonomaso, and Sakra Wonoo which are two of the facility indicators are also significant at 5%. Having primary level education, attitude of health attendants, and accessing the facility by car significantly (at 10%) influence the regularity of visits.

In determining the magnitude of influence of explanatory variables, marginal effect is introduced. At each level of a selected variable, holding all other independent variables equal, the predicted probabilities for regular visits to antenatal care facility during entire pregnancy (utilisation) are determined and the results are displayed in Table 3. From the findings, it comes out that the probability of regular number of visits to an antenatal care facility is likely to increase by approximately 0.041 if the age of the pregnant mother increases by a year. The probability of a regular visit to an antenatal care service facility by a pregnant mother is likely to change by 0.083 if the household size increases by 1 person. The marginal effect for occupational status suggests that for two individuals with an average age of 30.46 years and average household size of 3.83 persons, the predicted probability of a regular visit is around 0.113 more likely to occur for unemployed than for an employed pregnant mother.

The study also reveals that the change in probability for regular visits to an antenatal care facility decreases by 0.105 for pregnant mothers with primary education relative to those with middle/JHS educational level. The marginal effect shows that pregnant mothers with secondary/SHS educational status are 0.09 less likely to visit an antenatal care facility regularly than those with middle school/JHS education status. The change in probability of 26.5 percentage points indicates that the predicted probability of regular visits to an antenatal care facility is 0.265 lesser for a pregnant mother who is not satisfied than for the one who is satisfied with antenatal care service rendered at a health facility. Attitude of health staff is significant in determining the regularity of visits to antenatal healthcare by pregnant mothers. From Table 3, there will be a 6.4 percent increase in the regularity of visits when pregnant mothers perceive the attitude of health attendants as good as compared to those who perceive the attitude of caregivers as poor.

However, the means of the independent variables used in the logistic regression model are displayed in Table 2. Majority of respondents representing 39.5% came from the Mamponteng health facility. This was followed by respondents in Asonomaso hospital which had 33.5% with both Sakora Wonoo and Antoa represented by 27 respondents each indicating 13.5%. From Table 2, the mean age of the respondents was 28 years and majority of them were below 30 years. The study reveals that 187 respondents indicating 93.5% are married and 8 people representing 4% are separated and 5 respondents representing 2.5% are widowed. On the pattern of ANC utilisation, 161 women representing 80.5% utilised the service 4 times or more with 39 women utilising the service less than 4 times. Only 12 (2%) were traditionalists, 41.5% of respondents were Moslems, and the majority of 135 women were Christians. Most respondents (56.5%) utilised the service by walking followed by respondents who used the service by means of car (41.5%), whereas others used other means to using the service.

It is observed by the study that the likelihood of a pregnant mother paying regular visits to an antenatal care facility increases by 9.88 percent if the mother rates the quality of service rendered at the facility to be good as compared to rating the quality of service as poor. Again, it is observed that the probability of a regular visit to a health facility decreases by 11.17 percent and 15.72 percent, respectively, when a pregnant mother uses facilities at Asonomaso and Sakra Wonoo than when a pregnant mother uses the facility at Mamponteng. Again, the study reveals that using a car as a means of accessing the facility decreases the chances of regular visits by 6.70 percent as against walking to the health facility. This means that distance was important in determining the regularity or irregularity of visits made by pregnant mothers to health facilities to access antenatal care.

## 5. Discussion of Results

The purpose of this study was to investigate the influence of socioeconomic determinants on ANC use in peri-urban Ghana after the introduction of the free maternal health policy. The free maternal health policy was meant to significantly reduce the financial constraints that pregnant women go through in accessing healthcare services including ANCs with the view to reducing maternal mortality. Despite the effort of the government of Ghana on maternal healthcare, maternal mortality is still on the increase. Hence, there are calls by stakeholders to determine and explore beyond the surface to adopt the upstream approach to determine the factors responsible for maternal deaths. The socioeconomic determinants on the utilisation of ANC generally varies locally, nationally, and globally to include individual lifestyle choices, education, income, cost of care, social support systems, household size, government policies, social norms, and traditions. Hence, to improve ANC use, it is necessary and proper for stakeholders to go beyond the provision of free maternal services. In such a manner, the influences of ANC utilisation will be well explored to curtail avoidable maternal complications and mortalities. The results of the study, however, reveal that socioeconomic factors like age, household size, occupational status, secondary/SHS education, service satisfaction, service quality, and geographic distance significantly influence the use of ANC in peri-urban Ghana.

The age of the expectant mother influences the use of ANC services in peri-urban Ghana. The older the pregnant mother is, the more likely she is to use antenatal care service. The results in Table 3 show that the *z* statistic of the marginal effect of age is significant at 1%. This indicates that the probability of regular visits to antenatal care facilities will increase by 4.1 percent if the age of the pregnant mother increased by one year. The result is in agreement with the findings of Arthur [15], Joshi et al. [42], Godha et al. [43], and Ochako and Gichuhi [44] that the increase in age of a pregnant mother increases the use of ANC. There is the need to educate young mothers on the need to utilise maternal care services including ANC. The use of ANC increases with the increasing number of one's household. This could be due to the introduction of the free maternal health services including ANC provided for pregnant mothers. The policy aims to capture all pregnant women regardless of social class in order to reduce pregnancy complications and mortalities. It can be inferred that the increase in ANC visits with increase in age could be attributed to the fact that better health education is given to pregnant mothers during antenatal care sessions. The finding is consistent with the study of O'Meara et al. [45], Gudayu et al. [46], and Rai et al. [37] that household size of a pregnant mother significantly influences her ANC use.

Again, occupational status of a pregnant mother influences her quest of utilising maternal services including ANC in peri-urban Ghana. Although maternal services are supposed to be free in Ghana, yet pregnant mothers are constrained due to additional cost of care. These could either be direct or indirect as some of these costs are not absorbed by the free maternal healthcare policy. Among these are screening, laboratory tests, management of minor ailment, and immunisation. This makes pregnant women who are more resourced, more likely to afford and use such services and hinders the rates of utilisation by the less privileged, hence, not meeting the recommended visits by the WHO. It therefore, supports the study of Arthur [15] that wealth still influences the ANC use in Ghana even after the introduction of the free maternal health policy. This finding is, therefore, in agreement with works of Pandey and Singh [47], Saad-Haddad et al. [35], and Yadav and Kesarwani [5] that the occupational status of expectant mothers significantly influences their maternal care use.

Also, the multivariate results of the study show that satisfaction, physical distance, quality of service, education, and the attitude of health staff influence the rate expectant mothers use antenatal care service in peri-urban Ghana. Thus, improving the working ethics and standards of caregivers will contribute positively in the use of maternal health services including antenatal care use. This would help reduce mother-child mortality in the country. The introduction of the focused ANC by the study facilities should be well strengthened and monitored with the requisite logistics and protocols provided as recommended by the Johns Hopkins Program for International Education in Gynecology and Obstetrics (Jhpiego). Again, pregnant mothers should be well educated to know the importance of ANC services and to utilise such services. There is the need to use the mass media to sensitise women on the need to use ANC especially the young pregnant mothers as the results reveal that older women patronise ANC services more than their younger counterparts.

## 6. Conclusion

The study examined the socioeconomic determinants of antenatal care utilisation in peri-urban Ghana using data from four health facilities from the Kwabre East health directorate. The study hypothesised that socioeconomic factors like age, household size, physical location, service satisfaction, and quality of service do not influence the use of antenatal care after the introduction of the free maternal health services. Using multivariate analysis, the results indicate that most expectant mothers in peri-urban Ghana utilise the required visits for ANC services (80.5% for those attending 4+) as required by the WHO. Even though the free maternal health policy has appreciated the level of ANC use, sufficient utilisation has not yet been achieved as varied socioeconomic factors still pose problems to some pregnant mothers in using the service. It is, therefore, recommended that, to guarantee adequate use of antenatal service in peri-urban Ghana, upstream approaches like social support to ANC use should be provided to the less privileged women in addition to the policy. The protocol and logistics that prevail in health facilities should be streamlined and sustained to the benefit of the expectant mother. Hence, maternal services that are not absorbed by the policy should be reconsidered to help ease pregnant mothers of incurred costs in accessing ANC; adequate caregivers should be provided in facilities to complement the focused ANC care implemented by the health directorate. This will help increase the utilisation rate since mothers will spend less time accessing ANC and can make way for other productive services. There should also be a policy that emphasises on encouraging women education to at least the secondary/SHS level. There is the need to educate pregnant mothers especially the younger ones on the need to utilise maternal healthcare services including ANC so as to achieve the WHO minimum requirement of four visits on antenatal care and the sustainable development goals 3 and 5. This can be done through the various mediums of the mass media and during ANC sessions. Pregnant mothers who usually utilise ANC should also be encouraged to use the services to avert potential complications and mortalities.

## Figures and Tables

**Table 1:** 

Variables	Description	Expected sign
*X*_1_	Age of respondents	+
*X*_2_	Household size of respondents	+
*X*_3_	Occupational status of respondents	+
*X*_4_	Primary education	+, −
*X*_5_	Secondary/SHS education	+
*X*_6_	Vocational/technical education	−
*X*_7_	Tertiary education	+
*X*_8_	By car (as a means of utilising ANC)	+, −
*X*_9_	Others (any others means of utilising ANC)	−
*X*_10_	Asonomaso health centre	+
*X*_11_	SakraWonoo health centre	+
*X*_12_	Antoa health centre	−
*X*_13_	Choice of facility for ANC services	−
*X*_14_	Accessibility of ANC services	+, −
*X*_15_	Satisfaction of ANC services; 1 = satisfied, 0 = not satisfied	+
*X*_16_	Attitude of caregivers; 1 = satisfied, 0 = not satisfied	+, −
*X*_17_	Quality of service ANC; 1 = satisfied, 0 = not satisfied	+

**Table 2:** Socioeconomic characteristics of respondents on ANC use.

Variables	Frequency	Percentage (%)
Age distribution		
15–20	19	9.5
21–29	108	54.5
30–39	51	25.5
40–49	22	11
Mean household size	3.25	-
Employed	176	88
Unemployed	24	12
Had no formal education	12	6
Had primary education	10	5
Had middle/JHS education	60	30
Had secondary/SHS education	59	29.5
Had vocational/technical education	31	15.5
Had tertiary education	28	14
Had NHIS card	153	76.5
Did not have NHIS card	47	23.5
Mamponteng facility users	79	39.5
Asonomaso facility users	67	33.5
Sakrora Wonoo facility users	27	13.5
Antoa facility users	27	13.5

**Table 3:** Regression of ANC factors for the selected health facilities.

Dependent variable: utilisation (regular and irregular) dummy						
Independent Variables	Coef (*β*)	Std. Err.	*Z*	Marg. Eff. (*dy*/*dx*)	Std. Err.	*z*

*Socioeconomic characteristics *						
Age	1.42	0.38	3.71^*∗∗∗*^	0.04	0.01	8.22^*∗∗∗*^
HH size	2.83	1.01	2.81^*∗∗∗*^	0.08	0.02	3.62^*∗∗∗*^
Occupational status dummy	5.15	2.61	1.98^*∗∗*^	0.11	0.03	3.42^*∗∗∗*^
*Educational status*						
Primary	−4.31	2.36	−1.83^*∗*^	−0.10	0.05	−1.97^*∗∗*^
Secondary/SHS	−3.81	1.62	−2.35^*∗∗*^	−0.09	0.03	−2.81^*∗∗∗*^
Vocational/technical	−2.57	1.83	−1.40	−0.06	0.04	−1.42
Tertiary	−1.60	2.04	−0.78	−0.03	0.04	−0.78
*Geographical distance*						
By car	−2.53	1.49	−1.70^*∗*^	−0.07	0.03	−2.05^*∗∗*^
Others	−1.63	4.72	−0.35	−0.04	0.12	−0.37
*Facility*						
Asonomaso	−4.46	1.88	−2.37^*∗∗*^	−0.11	0.03	−3.47^*∗∗∗*^
Sakra Wonoo	−5.84	2.61	−2.23^*∗∗*^	−0.16	0.05	−2.87^*∗∗∗*^
Antoa	−0.60	2.02	−0.30	−0.01	0.04	−0.29
*Miscellaneous characteristics*						
Insurance status dummy	0.37	1.55	−0.24	−0.01	0.05	−0.24
Choice of facility dummy	−0.92	1.03	−0.89	−0.03	0.03	−0.94
Accessibility dummy	−1.49	1.44	−1.03	−0.04	0.04	−1.04
Satisfaction dummy	−8.51	2.68	−3.17^*∗∗∗*^	−0.27	0.04	−7.51^*∗∗∗*^
Attitude dummy	2.56	1.78	1.44	0.06	0.03	1.89^*∗*^
Quality of service dummy	4.30	1.83	2.35^*∗∗*^	0.10	0.04	3.72^*∗∗∗*^
*Constant*	−37.81	10.07	−3.75			
LR chi2(**19**) = 159.69^*∗∗∗*^						
Pseudo *R*2 = 0.81						
Log likelihood = −**18.83**						

*NB*. *∗*, *∗∗*, and *∗∗∗* represent significance at 10%, 5%, and 1%, respectively; *source*: *Field Survey, 2015. *
